# Real-World Effectiveness of Sorafenib versus Lenvatinib Combined with PD-1 Inhibitors in Unresectable Hepatocellular Carcinoma

**DOI:** 10.3390/cancers15030854

**Published:** 2023-01-30

**Authors:** Hsueh-Chien Chiang, Yang-Cheng Lee, Ting-Tsung Chang, Yih-Jyh Lin, Hung-Tsung Wu, Chung-Teng Wang, Chiung-Yu Chen, Po-Jun Chen, Ming-Tsung Hsieh, Sheng-Hsiang Lin, Shang-Hung Chen, Chiao-Hsiung Chuang, I-Chin Wu, Tzu-Chun Hong, Juei-Seng Wu, Meng-Zhi Han, Wei-Ting Chen, Chien-Ming Chiang, Kuan-Kai Hung, Hsin-Yu Kuo

**Affiliations:** 1 Department of Internal Medicine, National Cheng Kung University Hospital, College of Medicine, National Cheng Kung University, 138 Sheng Li Road, Tainan 70101, Taiwan; 2 Departments of Internal Medicine, Tainan Municipal Hospital, Tainan 70103, Taiwan; 3 Department of Surgery, National Cheng Kung University Hospital, College of Medicine, National Cheng Kung University, Tainan 70101, Taiwan; 4 Institute of Clinical Medicine, College of Medicine, National Cheng Kung University, Tainan 70101, Taiwan; 5 Department of Public Health, College of Medicine, National Cheng Kung University, Tainan 70101, Taiwan; 6 Biostatistics Consulting Center, National Cheng Kung University Hospital, College of Medicine, National Cheng Kung University, Tainan 70101, Taiwan; 7 Department of Oncology, National Cheng Kung University Hospital, College of Medicine, National Cheng Kung University, Tainan 70456, Taiwan; 8 National Institute of Cancer Research, National Health Research Institutes, Tainan 70456, Taiwan; 9 Department of Internal Medicine, An Nan Hospital, China Medical University, Tainan 70965, Taiwan

**Keywords:** immune checkpoint inhibitor, sorafenib, lenvatinib, hepatocellular carcinoma

## Abstract

**Simple Summary:**

Immunotherapy using immune checkpoint inhibitors (ICIs) (e.g., programmed cell death protein-1 (PD-1) inhibitors) combined with molecular targeted agents has been evaluated in clinical trials and has shown potential synergic effects and superior efficacy in unresectable hepatocellular carcinoma (uHCC). The optimal regimen for uHCC of combination therapy with a PD-1 inhibitor plus an MTKI remains controversial. A head-to-head comparison is still lacking regarding combination strategies involving the administration of PD-1 inhibitors with different MTKIs in uHCC. This highly original study evaluates the efficacy and safety of PD-1 inhibitors in combination with sorafenib or lenvatinib in a cohort of patients with uHCC. We observed that PD-1 inhibitors combined with lenvatinib resulted in more favorable survival outcomes without increased toxic effects compared with PD-1 inhibitors with sorafenib. Our data on efficacy and tolerability may enable clinicians to select optimal treatment strategies for HCC therapy.

**Abstract:**

Immune checkpoint inhibitors (ICIs) combined with multitarget tyrosine kinase inhibitors (MTKIs) exert a synergistic effect and are effective in unresectable hepatocellular carcinoma (uHCC). However, precise data regarding the real-world clinical applications of these combination therapies in uHCC are lacking. This study compared the treatment efficacy of sorafenib versus lenvatinib in combination with programmed cell death protein-1 (PD-1) inhibitors in patients with uHCC in a clinical setting. Among 208 patients with uHCC treated with PD-1 inhibitors, 88 were administered with ICIs in combination with sorafenib or lenvatinib. The treatment response and survival outcomes were evaluated. Predictors of survival were assessed by multivariate analysis. A total of 49 patients were treated with PD-1 inhibitors combined with sorafenib, and 39 patients were treated with PD-1 inhibitors combined with lenvatinib. The lenvatinib group exhibited a stronger objective response rate (ORR) (20.51% vs. 16.33%) and had a higher disease control rate (41.03% vs. 28.57%) than did the sorafenib group. The median overall survival was longer in the lenvatinib group than the sorafenib group (13.1 vs. 7.8 months; hazard ratio = 0.39, *p* = 0.017). The incidence of treatment-related adverse events was similar. PD-1 inhibitors combined with lenvatinib can be a feasible treatment strategy for HCC patients receiving MTKI-based combination therapy. PD-1 inhibitors combined with lenvatinib resulted in more favorable survival outcomes without increased toxic effects compared with PD-1 inhibitors with sorafenib. Additional larger-scale and prospective studies should be conducted to verify the study results.

## 1. Introduction

Hepatocellular carcinoma (HCC) is the third leading cause of cancer deaths worldwide [1,2]. Surgical resection is the standard treatment for patients with early-stage HCC with compensated liver function and is associated with a high five-year survival rate [3,4]. However, for unresectable HCC (uHCC), curative treatment strategies are not available, and the survival benefits of existing treatments are limited [5].

Raf-1 kinase and vascular endothelial growth factor (VEGF) receptor (VEGFR) mRNA are overexpressed in many HCC tumors [6]. In 2008, sorafenib, a multitarget tyrosine kinase inhibitor (MTKI), was reported to prolong median survival and time to progression by approximately three months in advanced HCC [7]. Subsequently, no breakthrough systemic therapy was developed until the discovery of another MTKI in 2018. Lenvatinib was reported to be non-inferior to sorafenib in terms of improvement in median overall survival (OS, 13.6 vs. 12.3 months), median progression-free survival (PFS, 7.4 vs. 3.7 months), median time to tumor progression (TTP, 8.9 vs. 3.7 months), and objective response rate (ORR, 24.1% vs. 9.2%) [8].

Immune checkpoint inhibitors (ICIs) were demonstrated to be beneficial in the treatment of various solid organ and hematological malignancies [9]. Programmed cell death protein-1 (PD-1) is overexpressed in HCC and enables a tumor to grow uncontrollably, leading to poor prognosis. PD-1 inhibitors, such as nivolumab and pembrolizumab, can enhance the immune reaction against tumor cells [10,11]. Nivolumab is a monoclonal antibody that inhibits the PD-1 receptor and exhibited an ORR of 20% in the dose-expansion phase and 15% in the dose-escalation phase [12]. Pembrolizumab is another monoclonal antibody that inhibits the PD-1 receptor and resulted in improved OS when administered as second-line therapy in patients with advanced HCC who were sorafenib-experienced [13].

Combination therapy targeting different mechanisms can be beneficial for patients with uHCC [14]. Because molecularly targeted drugs and immune checkpoint blockade govern different parts of the immune response, dual blockade of these factors may have a synergistic effect [15]. MTKIs significantly reduced the population of immunosuppressive tumor-associated macrophages and increased the infiltration of cytotoxic T lymphocytes [16,17]. Thus, molecularly targeted drugs can enhance antigen-presenting cell (APC) maturation and cytotoxic T-cell (CTL) activation and reduce immunosuppressive cell function. Moreover, immune checkpoint blockade can improve the antigen presentation from APC to CTL and reduce the exhaustion of CTLs, thus directly promoting tumor elimination. Compared with sorafenib, the combination of atezolizumab (an anti-PD-L1 antibody) and bevacizumab (an anti-VEGF antibody) more significantly improved OS and PFS in patients with uHCC [18]. Furthermore, a recent study demonstrated better efficacy and survival benefits of combining of a PD-1 inhibitor plus sorafenib compared with a PD-1 inhibitor alone [19].

The optimal regimen for HCC of combination therapy with a PD-1 inhibitor plus an MTKI remains controversial. Lenvatinib resulted in a better treatment response than sorafenib in terms of PFS, TTP, and ORR in the REFLECT study [8]. Scholars have reported that the combination of lenvatinib and ICIs was well-tolerated and had promising outcomes [20,21]. However, limited data are available for performing a head-to-head comparison of combination strategies involving the use of PD-1 inhibitors with different MTKIs in uHCC. Thus, this retrospective study compared the efficacy of various regimens containing a PD-1 inhibitor in combination with sorafenib and lenvatinib in a real-world cohort of patients with uHCC.

## 2. Materials and Methods

### 2.1. Study Design and Patients

**Figure 1:**
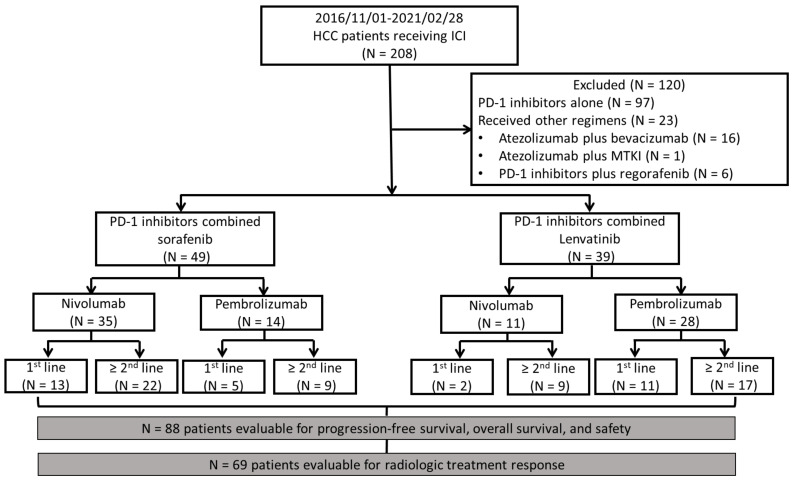
Flowchart for patient inclusion demonstrating the inclusion criteria, exclusion criteria, and number of patients in all the groups. Of the 19 patients not available for response assessment, 18 patients died before the radiologic evaluation and 1 patient was lost to follow-up. ICI, immune checkpoint inhibitor; MTKI, multitarget tyrosine kinase inhibitor; PD-1 inhibitor, programmed cell death protein-1 inhibitor.

This single-center, retrospective study was conducted at National Cheng Kung University Hospital, Tainan, Taiwan. Patients aged ≥18 years who had uHCC and received ICIs were included in the study. Each patient received an HCC diagnosis based on pathological or imaging findings according to American Association for the Study of Liver Diseases criteria [5]. Between 1 November 2016 and 28 February 2021, a total of 208 uHCC patients were treated with PD-1/PD-L1-targeting immunotherapy as systemic therapy. We excluded patients (N = 120) who received PD-1 inhibitors alone (N = 97), atezolizumab plus bevacizumab (N = 16), combination regimens containing ICI other than nivolumab or pembrolizumab (N = 1), or MTKIs other than sorafenib or lenvatinib (N = 6). After the exclusion of patients, the remaining patients were divided into the PD-1 inhibitors plus sorafenib and PD-1 inhibitors plus lenvatinib groups. Information on the sex, patients’ age, Eastern Cooperative Oncology Group (ECOG) performance status scale score, α-fetoprotein level, albumin–bilirubin (ALBI) grade, liver disease etiology, liver function, cancer stage, systemic line of combination therapy, and treatment modality was recorded. Figure 1 presents the study flowchart. The follow-up cutoff date was 30 April 2021.

This study was approved by the Institutional Review Board of National Cheng Kung University Hospital and validated in accordance with the ethical principles of the World Medical Association Declaration of Helsinki for medical research involving human participants (A-ER-109-199).

### 2.2. Assessment of Efficacy and Adverse Events

PD-1 inhibitors including nivolumab and pembrolizumab were investigated in this study. Nivolumab and pembrolizumab were administered intravenously at a dose of 3 mg/kg biweekly and 100–200 mg every 3 weeks, respectively.

Magnetic resonance imaging or triphase computed tomography was performed every 6 to 8 weeks. Two independent specialists examined changes in tumor size by using Response Evaluation Criteria in Solid Tumors (RECIST) version 1.1 [22] and the modified RECIST (mRECIST) [23] and categorized them into a complete response (CR), a partial response (PR), stable disease, or progressive disease (PD). Details regarding adverse events (AEs) were collected and examined in accordance with the National Cancer Institute’s Common Terminology Criteria for Adverse Events (version 5.0).

### 2.3. Endpoints

The primary outcomes of the study were the ORR, PFS, and OS. The ORR was defined as the percentage of patients who exhibited a CR or PR (according to mRECIST and RECIST, respectively) that was maintained for at least 28 days after the first demonstration of that rating based on an independent radiological review. PFS was the interval from the initiation of the PD-1 inhibitor in combination with an MTKI until the date of disease progression or death. OS was the interval from the initiation of the PD-1 inhibitor in combination with an MTKI until the date of death from any cause. The secondary endpoints were AE incidence and severity. Safety assessments included the documentation of AEs, clinical laboratory tests (hematologic and biochemical analyses), and physical examinations.

### 2.4. Statistical Analysis

The baseline characteristics of patients receiving PD-1 inhibitors combined with sorafenib or lenvatinib are shown as the median (interquartile range, IQR) or number (percentage). We used the paired sample *t*-test to determine differences between the sorafenib and lenvatinib groups. The primary endpoint of OS was evaluated using the Kaplan–Meier method with the log-rank test to determine the median and 95% confidence interval (CIs). To reduce the effect of confounding biases, we performed multivariate Cox regression analysis in which the follow-up period (in months) was included as the time variable. Death at the end of follow-up was set as the status variable. In addition to sorafenib versus lenvatinib, common variables associated with patient prognoses—age, sex, Child–Pugh score, Barcelona Clinic Liver Cancer classification, Cancer of the Liver Italian Program score, ECOG performance status scale score, ALBI grade, presence of distal metastasis, α-fetoprotein level, systemic drug, and PD-1 inhibitor type—were included in the model, and the “enter” method was used for variable selection. The 12 variables were treated as categorical variables. All tests were two-tailed with a *p*-value of <0.05 considered statistically significant.

Therapeutic efficacy, which was measured by the ORR, was compared among the patients with different characteristics by using Fisher’s exact test. A *p*-value of <0.05 was considered statistically significant. All analyses were conducted using SPSS software (SPSS Statistics 25.0, IBM Corp., Armonk, NY, USA).

## 3. Results

### 3.1. Differences in Baseline Characteristics between the Sorafenib and Lenvatinib Groups

Of the 88 patients with uHCC included in this study, 49 were treated with PD-1 inhibitors plus sorafenib and 39 were administered PD-1 inhibitors plus lenvatinib. The baseline characteristics were balanced between the sorafenib and lenvatinib groups (Table 1).

The median dosages of sorafenib and lenvatinib administered orally were 400 mg (IQR, 400–800 mg) per day and 8 mg (IQR, 8–10 mg) per day. The median duration of combination therapy was 73 (IQR, 43–168) days in the sorafenib group and 70 (IQR, 57–136) days in the lenvatinib group.

### 3.2. Differences in the ORR between the Sorafenib and Lenvatinib Groups

Table 2 presents the treatment responses of the patients in accordance with mRECIST and RECIST. In total, 35 patients in the sorafenib group and 34 patients in the lenvatinib group had at least one follow-up image and were therefore assessable for tumor response evaluation. Of the 19 patients not available for response assessment, 18 patients died before the radiologic evaluation and 1 patient was lost to follow-up. On the basis of mRECIST, 9 (18.37%) and 9 (23.08%) patients in the sorafenib and lenvatinib groups, respectively, achieved an objective response (*p* = 0.944). On the basis of RECIST, 8 (16.33%) and 8 (20.51%) patients in the sorafenib and lenvatinib groups, respectively, achieved an objective response (*p* = 0.948).

Table S1 presents the results of subgroup analysis performed on the basis of the systemic line of combination therapy of PD-1 inhibitors plus MTKIs. In the first-line combination therapy group, 4 (26.67%) and 3 (30.00%) patients in the sorafenib and lenvatinib groups, respectively, achieved an objective response (*p* = 0.863). In the second- and further-line combination therapy groups, 5 (25.00%) and 6 (25.00%) patients in the sorafenib and lenvatinib groups, respectively, achieved an objective response (*p* = 1.000).

### 3.3. Survival Outcomes in the Sorafenib and Lenvatinib Groups

**Figure 2:**
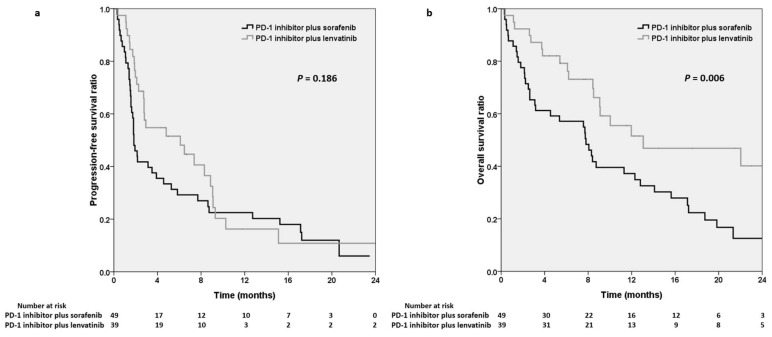
Kaplan–Meier analysis demonstrating progression-free survival (**a**) and overall survival (**b**) for the PD-1 inhibitors plus sorafenib and the PD-1 inhibitors plus lenvatinib. PD-1, programmed cell death protein-1.

The median PFS of the sorafenib and lenvatinib groups was 1.8 and 6.1 months, respectively (*p* = 0.186; Figure 2a). The median OS of the sorafenib and lenvatinib groups was 7.8 and 13.1 months, respectively (*p* = 0.006; Figure 2b).

### 3.4. OS According to ALBI Grade and Systemic Line of Combination Therapy

**Figure 3:**
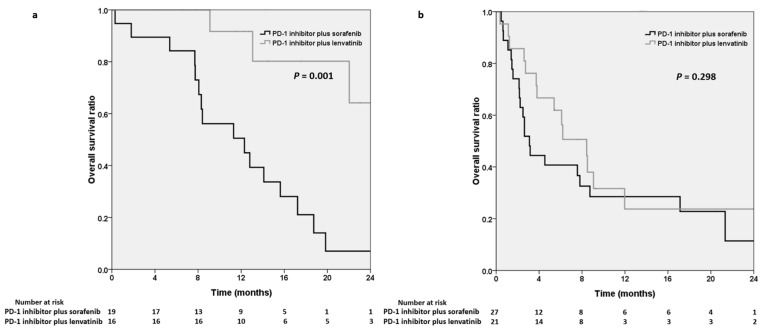
Kaplan–Meier analysis illustrating overall survival in the PD-1 inhibitors plus sorafenib group and the PD-1 inhibitors plus lenvatinib group in patients with albumin–bilirubin (ALBI) grade 1 (**a**) and ALBI grade 2 or 3 (**b**), respectively. PD-1, programmed cell death protein-1.

We performed survival analysis on the basis of ALBI grade and the systemic line of combination therapy. In subgroup analysis, we divided the cohort into two groups based on the patients’ baseline ALBI grade. A total of 35 and 48 patients had an ALBI grade of 1 and of 2 or 3, respectively (Figure 3a,b). The baseline ALBI grade was not recorded for 5 patients. The Kaplan–Meier curve indicated that the OS was shorter in the sorafenib group (12.3 months) than in the lenvatinib group (median OS not reached, *p* = 0.001) in the patients with ALBI grade 1 (Figure 3a).

**Figure 4:**
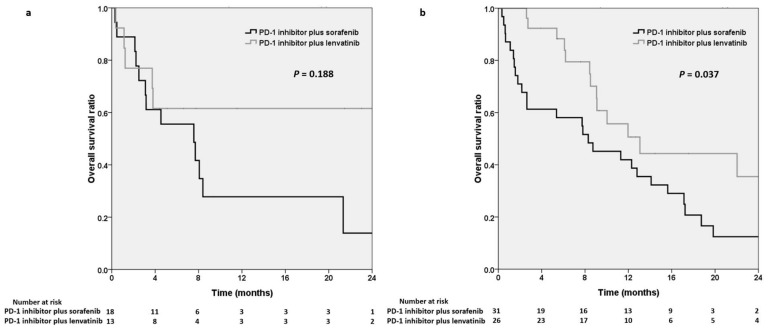
Kaplan–Meier analysis demonstrating overall survival in the PD-1 inhibitors plus sorafenib group and the PD-1 inhibitors plus lenvatinib group in patients receiving combination therapy as first-line treatment (**a**) and as second- or further-line treatment (**b**), respectively. PD-1, programmed cell death protein-1; MTKI, multitarget tyrosine kinase inhibitor.

Regarding the systemic line of combination therapy, we noted slightly longer OS in the lenvatinib group (median OS not reached) than in the sorafenib group (7.6 months, *p* = 0.188) in the patients who received an ICI plus an MTKI as first-line therapy (Figure 4a). By contrast, OS was significantly longer in the lenvatinib group than in the sorafenib group (13.1 vs. 8.3 months, *p* = 0.037; Figure 4b) in the patients receiving an ICI plus an MTKI as second- or further-line therapy.

### 3.5. Prognostic Factors for Survival

Univariate and multivariate analyses were performed to identify the determinants of OS (Table 3). The results of multivariate Cox regression analysis revealed that poor performance status, poor liver function reserve (Child–Pugh B or C), and combination with sorafenib were unfavorable prognostic factors for survival.

The hazard ratio (HR) of the patients receiving a combination of ICIs plus lenvatinib compared with those receiving ICIs plus sorafenib was 0.39 (95% CI, 0.18–0.85; *p* = 0.017) for OS.

### 3.6. Incidence of Treatment-Related AEs between the Sorafenib and Lenvatinib Groups

Table S2 lists AEs observed in both groups. A total of 16 (32.65%) and 10 (25.64%) patients in the sorafenib and lenvatinib groups, respectively, experienced AEs. The incidence was similar in the two groups (*p* = 0.480). Concerning the severity of AEs, one (2.04%) patient in the sorafenib group had severe AEs (grade 3–4) and one (2.56%) patient in the lenvatinib group had severe AEs (grade 3–4). The incidence of severe AEs was similar in the two groups (*p* = 0.872).

## 4. Discussion

To the best of our knowledge, this is the first study to compare therapeutic outcomes and safety profiles between different MTKI-based therapies combined with PD-1 inhibitors in patients with uHCC. In this real-world cohort study, a potential higher treatment response was noted for lenvatinib combined with ICIs compared with PD-1 inhibitors combined with sorafenib in the patients with uHCC. PD-1 inhibitors combined with lenvatinib resulted in longer OS than did PD-1 inhibitors combined with sorafenib in the patients with uHCC, especially those with preserved liver function and those receiving the combination therapy of PD-1 inhibitors and MTKIs as second- or further-line therapy. Moreover, the safety profile was comparable for these combination therapies. Differences in efficacy among different MTKI-based regimens can help clinicians to select the most appropriate drug for cancer management in HCC patients.

In combination therapy, VEGF inhibitors increase intratumoral infiltration and cytotoxic T lymphocyte survival, resulting in a favorable immune microenvironment for the antitumoral activity of PD-1 inhibitors, which may exert a synergistic effect during combination therapy [24,25]. Sorafenib targets Raf serine/threonine kinases (Raf-1, wild-type B-Raf, and oncogenic B-Raf V600E) and VEGFR 1–3 to inhibit tumorigenesis and tumor progression [6]. Lenvatinib is a small-molecule tyrosine kinase inhibitor that inhibits VEGFR 1–3 and fibroblast growth factor receptors (FGFRs) 1–4 [26]. Furthermore, lenvatinib directly acts on tumor-infiltrating lymphocytes by reducing Treg differentiation to improve anti-PD-1 efficacy by blocking FGFR 4 [27]. Compared with sorafenib, lenvatinib may exert a stronger synergistic effect in combination therapy. However, data on the efficacy of MTKIs in combination with PD-1 inhibitors are scant. In the present real-world analysis, combination therapy with lenvatinib and PD-1 inhibitors resulted in longer OS than did sorafenib use in combination therapy. Moreover, combination therapy involving lenvatinib improved OS (HR, 0.39; 95% CI, 0.18–0.85, *p* = 0.017) in the patients receiving combination therapy with ICIs and MTKIs. Thus, lenvatinib combined with nivolumab or pembrolizumab may serve as a therapeutic regimen for HCC patients receiving MTKI-based combination therapy.

Many combination therapies are being developed, and anti-PD-1 agents in combination with anti-angiogenic targeted therapies have resulted in a favorable response rate [28]. However, published biomarker data that can provide guidance for the selection of ICIs or MTKIs for HCC treatments are limited. An important unmet therapeutic need is to identify the most effective ICI–antiangiogenic agent combination. Huang et al. reported that the combination of lenvatinib and PD-1 inhibitors resulted in a 25.9% objective response rate in 29 advanced HCC patients on the basis of RECIST. The 12-month OS rate was 53.7% [20]. Chen et al. reported that the objective response was 22.4% in patients with HCC who received sorafenib plus PD-1 inhibitors [19]. However, no head-to-head comparison study has examined differences in efficacy between sorafenib and lenvatinib in combination with PD-1 inhibitors. In this study, higher ORRs and disease control rates were observed in the patients who received lenvatinib with PD-1 inhibitors than in those who received sorafenib with PD-1 inhibitors; however, this difference was nonsignificant. Furthermore, longer PFS was observed in the patients receiving PD-1 inhibitors plus lenvatinib (6.1 vs. 1.8 months, *p* = 0.186). As presented in Figure 2a, although the Kaplan–Meier curves crossed in the long run, ICI plus lenvatinib appeared to have higher efficacy in the first eight months. This may have been due to the small number of patients enrolled in the study, the imbalance in the patient numbers, or the heterogeneity of the patient population between the sorafenib and lenvatinib groups.

In our subgroup analysis (Figure 3), we noted that the survival benefit of PD-1 inhibitors plus lenvatinib was more prominent in the patients with ALBI grade 1, who had better-preserved liver function. Well-preserved liver function may lead to better outcomes [2], and HCC patients with well-preserved liver function could have more treatment options [29]. In patients with preserved liver function, combination therapy with PD-1 inhibitors plus lenvatinib resulted in more favorable survival outcomes than PD-1 inhibitors plus sorafenib did. The presence of differences between these agents may guide clinical management in patients with preserved liver function and for those receiving combination therapies. Advancements in the development of combination therapies with synergistic effects and biomarkers for identifying the optimal patients will be important tasks in the future.

Emerging evidence has suggested a better treatment response by combination therapy, due to the synergistic effects, as compared to single agents in HCC treatment [21]. In this study, only patients treated with a combination of PD-1 inhibitors and MTKIs were enrolled. Hence, we performed survival analyses for patients, including 88 who had originally enrolled in the analyses, and 97 patients administered with PD-1 inhibitors alone. The median OS was significantly longer in the PD-1 inhibitors combined with lenvatinib group than PD-1 inhibitors use alone (13.1 vs. 6.0 months, *p* = 0.008). The findings were similar to those of a previous study, showing that combination of anti-PD-1 and MTKIs could provide potentially synergistic effects that render long-term survival possible [19]. Further investigation to determine predictors of good responders and to discover more effective combinatorial regimens should provide more personalized immunotherapies.

The major limitation of our study is its small sample. Moreover, a retrospective study may be affected by missing data, disordered data, and ambiguous timing of the sequence of treatments. Second, various treatment options can be applied in a single patient because of changing HCC status before ICI administration, including several sessions of radiofrequency ablation interspersed with several instances of transarterial chemoembolization. Complicated treatment strategies may affect residual liver function and the efficacy of PD-1 inhibitors, such as changes in the microenvironment after radiofrequency ablation and transarterial chemoembolization. Furthermore, different treatment modalities administered after disease progression or treatment discontinuation may affect survival and treatment outcomes. Future prospective studies are warranted to evaluate the results of the current study.

## 5. Conclusions

In conclusion, lenvatinib combined with nivolumab or pembrolizumab can be an effective treatment option and does not strengthen toxic effects compared with sorafenib-based combination therapy in patients with uHCC. Additional larger-scale prospective studies should be conducted to verify the results of the current study.

## Figures and Tables

**Table 1:** Baseline characteristics of all 88 HCC patients who received immune checkpoint inhibitors combined with multitarget tyrosine kinase inhibitors.

Characteristic	PD-1 Inhibitors + Sorafenib (N = 49)	PD-1 Inhibitors + Lenvatinib (N = 39)	*p*-Value
	Number (%)	Number (%)	
**Gender**			
Female	10 (20.41)	8 (20.51)	0.990
Male	39 (79.59)	31 (79.49)	
**Age, years—median (IQR)**	60.0 (53.0–65.0)	65.0 (54.0–71.0)	0.608
<55 years	14 (28.57)	10 (25.64)	0.762
≥55 years	35 (71.43)	29 (74.36)	
**α-Fetoprotein, ng/mL ^†^**			
<400 ng/mL	24 (48.98)	22 (56.41)	0.472
≥400 ng/mL	24 (48.98)	16 (41.03)	
**Etiology of chronic liver disease**			
No liver disease	4 (8.16)	2 (5.13)	0.542
Liver disease present	45 (91.84)	36 (94.87)	
Chronic hepatitis B	31 (63.27)	30 (76.92)	0.293
Chronic hepatitis C	13 (26.53)	8 (20.51)	0.443
Alcoholic hepatitis	4 (8.16)	2 (5.13)	0.542
Nonalcoholic steatohepatitis	0 (0)	1 (2.56)	0.323
**Child-Pugh class**			
A	32 (65.31)	27 (69.23)	0.701
B–C	17 (34.69)	12 (30.77)	
**BCLC stage**			
B	5 (10.20)	6 (15.38)	0.471
C–D	44 (89.80)	33 (84.62)	
**CLIP**			
0–1	16 (32.65)	18 (46.15)	0.231
2–5	32 (65.31)	21 (53.85)	
**Distant metastases**			
No	22 (44.90)	14 (35.90)	0.399
Yes	27 (55.10)	25 (64.10)	
**ALBI grade ^‡^**			
Grade 1	19 (39.58)	16 (41.03)	0.861
Grade 2–3	27 (56.25)	21 (53.85)	
**ECOG**			
Score 0	25 (51.02)	22 (56.41)	0.808
Score ≥ 1	20 (40.82)	17 (43.59)	
**Combination therapy as systemic line**			
1st line	18 (36.73)	13 (33.33)	0.744
≥2nd line	31 (63.27)	26 (66.67)	
**PD-1 inhibitors types**			
Nivolumab	36 (73.47)	13 (33.33)	<0.001
Pembrolizumab	15 (30.61)	26 (66.67)	
**PD-1 inhibitors cycles**			
Median (IQR)	6 (4–11)	6 (4–8)	0.567
**PD-1 inhibitors total dose (mg)**			
Median (IQR)	160 (100–200)	600 (450–1095)	0.390
**MTKI dose (mg/day)**			
Median (IQR)	400 (40 –700)	8 (8–10)	<0.001
**MTKI duration (day)**			
Median (IQR)	73 (43–168)	70 (57–136)	0.777

PD-1 inhibitors, programmed cell death protein-1 inhibitors; IQR, interquartile range; CLIP, Cancer of the Liver Italian Program Scoring System; BCLC, Barcelona Clinic Liver Cancer; ALBI, albumin–bilirubin; ECOG, Eastern Cooperative Oncology Group performance status scale; MTKIs, multitarget tyrosine kinase inhibitors. ^†^ 1 patient treated with PD-1 inhibitors plus sorafenib and 1 patient treated with PD-1 inhibitor plus lenvatinib did not have α-Fetoprotein data. ^‡^ 3 patients treated with PD-1 inhibitors plus sorafenib and 1 patient treated with PD-1 inhibitor plus lenvatinib did not have ALBI grade.

**Table 2:** Treatment response between patients treated with immune checkpoint inhibitors combined with multitarget tyrosine kinase inhibitors.

	PD-1 Inhibitors + Sorafenib (N = 49) ^†^	PD-1 Inhibitors + Lenvatinib (N = 39) ^‡^	*p*	PD-1 Inhibitors + Sorafenib (N = 49) ^†^	PD-1 Inhibitors + Lenvatinib (N = 39) ^‡^	
	mRECISTN (%)	mRECISTN (%)		RECISTN (%)	RECISTN (%)	*p*
**Response**			0.827			0.703
CR	2 (4.08)	1 (2.56)		0 (0)	0 (0)	
PR	7 (14.29)	8 (20.51)		8 (16.33)	8 (20.51)	
SD	5 (10.20)	7 (17.95)		6 (12.24)	8 (20.51)	
PD	21 (42.86)	18 (46.15)		21 (42.86)	18 (46.15)	
Not evaluable	14 (28.57)	5 (12.82)		14 (28.57)	5 (12.82)	
ORR	9 (18.37)	9 (23.08)	0.944	8 (16.33)	8 (20.51)	0.948
DCR	14 (28.57)	16 (41.03)	0.561	14 (28.57)	16 (41.03)	0.561

PD-1 inhibitors, programmed cell death protein-1 inhibitors; CR, complete response; PR, partial response; SD, stable disease; PD, progressive disease; ORR, objective response rate; DCR, disease control rate; RECIST, Response Evaluation Criteria in Solid Tumors; mRECIST, modified Response Evaluation Criteria in Solid Tumors. ^†^ Of the 14 subjects not available for response assessment, 13 patients died before the first radiologic evaluation and 1 patient was lost to follow-up. ^‡^ Of the 5 subjects not available for response assessment, 5 patients died before the first radiologic evaluation.

**Table 3:** The multivariate Cox regression analysis of factors predicting overall survival.

Factors	Case No.	HR	95% CI	*p*-Value
**Age (year)**				
≤55 vs. >55	24/64	0.906	0.453–1.811	0.780
**Gender**				
male vs. female	70/18	1.715	0.792–3.716	0.171
**Child-Pugh score**				
A vs. B–C	59/28	0.144	0.056–0.370	<0.001
**BCLC stage**				
B vs. C–D	11/77	1.416	0.536–3.745	0.483
**CLIP score**				
0-1 vs. 2-5	35/53	0.786	0.317–1.945	0.602
**ECOG score**				
0 vs. ≥ 1	47/38	0.296	0.135–0.651	0.002
**ALBI grade ^†^**				
1 vs. 2–3	35/48	0.539	0.198–1.466	0.226
**Distal metastasis**				
positive vs. negative	52/36	1.713	0.841–3.488	0.138
**α-Fetoprotein level (ng/mL) ^‡^**				
< 400 vs. ≥ 400	46/40	0.921	0.457–1.857	0.819
**Combination therapy as systemic line**				
1st line vs. ≥ 2nd line	31/57	1.312	0.660–2.606	0.439
**MTKI type**				
lenvatinib vs. sorafenib	49/39	0.394	0.183–0.849	0.017
**ICI type ^§^**				
nivolumab vs. pembrolizumab	47/38	1.213	0.576–2.557	0.611

HR, hazard ratio; CI, confidence interval; CLIP, Cancer of the Liver Italian Program Scoring System; BCLC, Barcelona Clinic Liver Cancer; ECOG, Eastern Cooperative Oncology Group performance status scale; ALBI, albumin–bilirubin; MTKIs, multitarget tyrosine kinase inhibitors; ICIs, immune checkpoint inhibitors. ^†^ Five patients had no baseline ALBI grade data. ^‡^ Two patients had no baseline α-Fetoprotein level. ^§^ Three patients had accepted both nivolumab and pembrolizumab during combination therapy.

## Data Availability

All datasets of the current study are available from the corresponding author on reasonable request.

## Supplementary Materials

The following supporting information can be downloaded at: https://www.mdpi.com/article/10.3390/cancers15030854/s1, Table S1. Treatment response between patients treated with PD-1 inhibitors combined with multitarget tyrosine kinase inhibitors according to systemic line of combination therapy; Table S2. Adverse events among the patients treated with PD-1 inhibitors combined with sorafenib and PD-1 inhibitors combined with lenvatinib.

## Abbreviations

AE	Adverse events
ALBI	Albumin–bilirubin
BCLC	Barcelona Clinic Liver Cancer
CI	Confidence intervals
CLIP	Cancer of the Liver Italian Program
CR	Complete response
CTCAE	Common Terminology Criteria for Adverse Events
ICI	Immune checkpoint inhibitors
MTKI	Multitarget tyrosine kinase inhibitor
ORR	Objective response rates
OS	Overall survival
PD	Progressive disease
PR	Partial response
SD	Stable disease
VEGF	Vascular endothelial growth factor
