# Supplementary material for: Real-World Effectiveness of Sorafenib versus Lenvatinib Combined with PD-1 Inhibitors in Unresectable Hepatocellular Carcinoma
Source: Cancers (Basel). 2023 Jan 30;15(3):854. doi: 10.3390/cancers15030854 (PMC9913272; doi:10.3390/cancers15030854)
Supplement: Supplementary file 1 [file cancers-15-00854-s001.zip › cancers-2157694-supplementary.pdf]

Table S1. Treatment response between patients treated with PD-1 inhibitors combined multitarget tyrosine kinase inhibitors according to systemic line of combination therapy

	Systemic therapy line of PD-1 inhibitors plus MTKI					
	1st line			≥ 2nd line		
	Anti-PD-1 + sorafenib (N = 18)	Anti-PD-1+ lenvatinib (N = 13)		Anti-PD-1 + sorafenib (N = 31)	Anti-PD-1 + lenvatinib (N = 26)	
	mRECIST N (%)	mRECIST N (%)	*p*	mRECIST N (%)	mRECIST N (%)	*p*
**Response**			0.936			0.752
CR	1 (6.67)	0 (0)		1 (5.00)	1 (4.17)	
PR	3 (20.00)	3 (30.00)		4 (20.00)	5 (20.83)	
SD	2 (13.33)	1 (10.00)		3 (15.00)	6 (25.00)	
PD	9 (60.00)	6 (60.00)		12 (60.00)	12 (50.00)	
NE	3 (16.6)	3 (23.08)		11 (35.48)	2 (7.69)	
ORR	4 (26.67)	3 (30.00)	0.863	5 (25.00)	6 (25.00)	1.000
DCR	6 (40.00)	4 (40.00)	1.000	8 (40.00)	12 (50.00)	0.518

PD-1 inhibitors, programmed cell death protein-1 inhibitors; MTKI, multitarget tyrosine kinase inhibitors; CR, complete response; PR, partial response; SD, stable disease; PD, progressive disease; NE, not evaluable; ORR, objective response rate; DCR, disease control rate; mRECIST, modified Response Evaluation Criteria in Solid Tumors

Table S2. Adverse events among the patients treated with PD-1 inhibitors combined sorafenib and PD-1 inhibitors combined lenvatinib

	PD-1 inhibitors + sorafenib (N = 49)	PD-1 inhibitors + lenvatinib (N = 39)	*p*
**Side effect**			
No	33 (67.34)	29 (74.36)	0.480
Yes	16 (32.65)	10 (25.64)	
**Side effect type**			
Fatigue or weakness	1 (2.04)	2 (5.13)	
Gastrointestinal			
Nausea or vomit or dyspepsia or anorexia	4 (8.16)	0 (0)	
Diarrhea or colitis	2 (4.08)	0 (0)	
Inflammatory			
Hepatitis	2 (4.08)	2 (5.13)	
Skin rash	8 (16.32)	4 (10.26)	
Pneumonitis	0 (0)	0 (0)	
Arthritis	0 (0)	1 (2.56)	
Endocrine			
Diabetes mellitus	0 (0)	0 (0)	
Hypophysitis with adrenal insufficiency	0 (0)	0 (0)	
Hyperthyroidism	1 (2.04)	0 (0)	
Hypothyroidism	1 (2.04)	1 (2.56)	
**Side effect grade**			
1	9 (18.37)	6 (15.38)	0.866
2	6 (12.24)	3 (7.69)	
3	1 (2.04)	1 (2.56)	
4	0 (0)	0 (0)	
**Side effect grade**			
1 & 2	9 (18.37)	15 (38.46)	0.730
3 & 4	1 (2.04)	1 (2.56)	0.872

PD-1 inhibitors, programmed cell death protein-1 inhibitors
